# Serum amyloid A1 (SAA1) protein in human colostrum

**DOI:** 10.1002/2211-5463.12383

**Published:** 2018-01-31

**Authors:** George H. Sack, Natasha Zachara, Nadine Rosenblum, C. Conover Talbot, Simion Kreimer, Robert Cole, Thomas L. McDonald

**Affiliations:** ^1^ Department of Biological Chemistry Johns Hopkins University School of Medicine Baltimore MD USA; ^2^ Department of Medicine Johns Hopkins University School of Medicine Baltimore MD USA; ^3^ Department of Obstetrics and Gynecology Johns Hopkins University School of Medicine Baltimore MD USA; ^4^ Institute for Basic Biomedical Sciences Johns Hopkins University School of Medicine Baltimore MD USA; ^5^ Department of Pathology University of Nebraska Medical Center Omaha NE USA

**Keywords:** acute‐phase, amyloid, colostrum, serum amyloid A

## Abstract

Proteins of the serum amyloid A (SAA) family have been remarkably conserved in evolution. Their biologic function(s) are not fully defined but they are likely to be a part of primordial host defense. We have detected a ∼ 12‐kDa protein reacting with antibodies against serum amyloid A (SAA) in human colostrum by western blotting. Mass spectrometry identified the reactive species as SAA1, previously identified as a prominent member of the acute‐phase response in serum. Our finding SAA1 in human colostrum contrasts with bovine, caprine and ovine colostrum where a species corresponding to putative SAA3 is uniformly present. SAA1 protein in human colostrum presumably contributes to neonatal protection.

**Abbreviations**
GRE: glucocorticoid responsive element
LPS: lipopolysaccharide
RACE: rapid amplification of cDNA ends
SAA: serum amyloid A
sfb: segmented filamentous bacteria
UTR: untranslated region

Human serum amyloid A (SAA) proteins constitute a family of proteins with genes encoded on a small region of chromosome 11p15.1 1, 2, syntenic to murine chromosome 7 3. SAA gene and protein sequences are very well‐conserved in mammals as well as in less complex organisms 4, 5. SAA was initially identified as the parent serum protein of polypeptides that associate with insoluble fibrillary deposits (called ‘amyloid’) in ‘secondary’ amyloidosis 6, 7, 8. Elevated serum levels of SAA proteins and the likelihood of developing amyloidosis are generally associated with inflammatory conditions during which there is enhanced SAA transcription and translation (generally in response to cytokines including IL‐1, IL‐6 and TNF). SAA1 and SAA2 along with C‐reactive protein are the most prominent members of the stereotyped acute‐phase response (APR) 9.

In humans, two nearly identical genes encoding 104 aa serum proteins (SAA1 and SAA2) are arranged in opposing transcriptional orientations 10 with conservative aa polymorphisms recognized between them. Another gene, SAA4, is telomeric to SAA2 and appears to be translated constitutively at low levels 11, 12. A third locus, currently termed SAA3p, is 90 kb telomeric to SAA4. Earlier sequence data from a genomic clone for SAA3 (then called GSAA1) predicted a protein with 104 aa but with prominent differences in the amino‐terminal region 13. To date, the human SAA3 protein has not been identified in tissues or serum.

In 2001, McDonald *et al*. 14 found a highly abundant protein with a sequence similar to that predicted for SAA3 in bovine, equine and ovine colostrum. Common to all isolates was the aa sequence TFLK found within the first eight amino acids of the amino‐terminal region of the secreted proteins 15. Cloning and sequencing the corresponding bovine cDNA revealed a sequence corresponding to a 113 residue mature protein 14. Larson *et al*. 16 extended this study to human mammary gland epithelial cells. Following stimulation with prolactin or lipopolysaccharide (LPS), transcription was detected by RT‐PCR and sequencing predicted a 42 aa protein matching the amino‐terminal region previously predicted for SAA3p (formerly called GSAA1–13). These and other observations disclosed a single nt change in the human SAA3p gene leading to a premature stop codon. To date, neither the 42 aa nor other related species has been identified in humans.

More recently, Knee *et al*. 17 used an ELISA to measure SAA protein(s) in colostrum and breast milk of women following preterm delivery. Concentrations were highest (mean 4.1 μg·mL^−1^) on the first postpartum day and fell to a mean of 75 ng·mL^−1^ by day 7. Levels were not affected by the presence or absence of chorioamnionitis. Although SAA3 was proposed to be the reactive species, details including the molecular weight and aa sequence(s) responsible were not determined.

We sought to verify the presence and identity of SAA species in human colostrum by both antibody reactivity and direct protein sequencing.

## Materials and methods

Samples of human colostrum (2–4 mL) were collected by one of the authors (N.R.) 1–3 days postpartum during routine clinical lactation care following informed consent in accordance with approved Johns Hopkins IRB protocol #IRB00053221. Once obtained, specimens were handled anonymously, promptly placed on ice and then stored at −20 °C.

### Sample preparation

A quantity of 75 μL of thawed, vortexed sample was mixed with 75 μL of PBS and placed on ice for 10 min. The sample was then spun at 16 000 ***g*** for 10 min at 4 °C leading to a firm pellet, a tenacious superficial upper layer and an intervening clear layer. The clear layer was transferred to a fresh tube and eight volumes of 10% trichloroacetic acid in acetone (−20 °C) was added. The tube was mixed by vortex and a large white precipitate formed. The sample was placed at −20 °C for 4 h and then centrifuged for 10 min at 4 °C; a firm pellet formed. The supernatant was removed without disturbing the pellet and eight volumes of acetone at −20 °C was added. The tube was vortexed briefly and then placed on ice for 10 min. The supernatant was removed and the pellet was resuspended in a net of 200 μL of 20 mm ammonium bicarbonate, pH 7.0. The pellet itself was tenacious and gradually the protein level in the resuspension buffer rose. The resuspended sample was kept at 4 °C. Protein concentrations were determined using 1 : 200 dilutions and the BCA system (ThermoFisher, Waltham, MA, USA).

SDS gel electrophoresis was performed using 18% Novex Tris‐glycine (LifeTechnologies, Carlsbad, CA, USA) gels for 1 h at 190 V in buffer containing 25 mm Tris, 192 mm glycine, 0.1% SDS. Molecular weight standards (Bio‐Rad, Hercules, CA, USA, #161‐0374) were included. Gel contents were transferred to poly(vinylidene difluoride) (PVDF) membranes for 2 h at 50 V in buffer containing 25 mm Tris, 50 mm glycine, 10% methanol. A parallel set of gel lanes was stained for protein using Coomassie brilliant blue. Other gels were stained with SYPRO Ruby (Invitrogen, Waltham, MA, USA) in an effort to detect low‐molecular weight species.

The PVDF transfer membrane was washed in 50 mm Tris, 150 mm NaCl, 0.05% Tween 20 (TBST) for 1 h at room temperature. Next, the membrane was placed in TBST with 3% milk for 1 h at room temperature and then washed again in TBST for an additional hour. The primary antibody (Rat anti‐SAA monoclonal antibody #C‐1007 Tridelta Development Ltd, Maynooth, Ireland) in 3% milk/TBST was added and the membrane was gently agitated for 16 h at 4 °C. The membrane was then washed in TBST for an hour at room temperature. The secondary antibody (peroxidase‐conjugated goat anti‐rat IgG [H+L]; Invitrogen #62‐9520) in 3% milk/TBST was added and the membrane was incubated at room temperature for 1 h and then washed in TBST for an additional hour at room temperature. Imaging used an Amersham 600 imager and Millipore chemiluminescent substrates.

For amino acid sequence determination, an aliquot of the original frozen specimen was diluted 1 : 1 with 137 mm NaCl, 10 mm phosphate, pH 7.4 and subjected to SDS electrophoresis as noted above. The gel was stained with Coomassie Brilliant Blue and bands of appropriate molecular weight were excised using a sterile blade and placed in tubes, previously washed in HPLC‐grade methanol and acetonitrile. The samples were reduced with DTT, alkylated with iodoacetamide, and digested in‐gel with trypsin and Lys‐C overnight. Polypeptides were extracted and analyzed on the Orbitrap Fusion using a 90‐min gradient.

DNA and protein sequence data were obtained and compared using the UCSG Genome browser for the human and other mammalian DNA sequence databases, December 2013 (GRCh38/hhg19) assembly.

## Results

**Figure 1:**
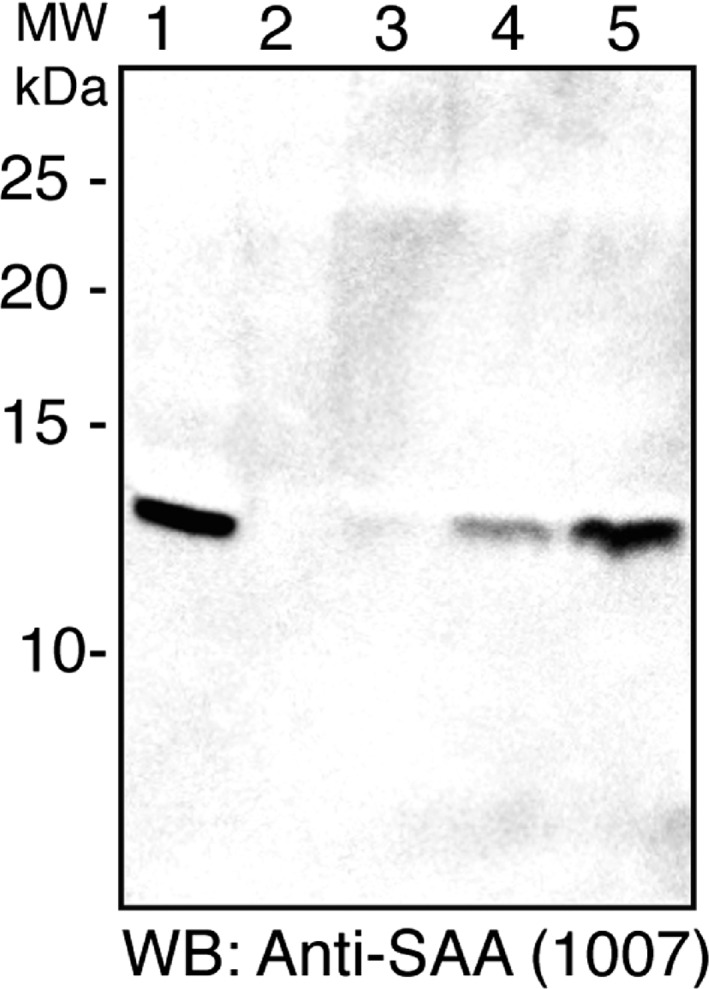
Western blot showing reactivity of five human colostrum samples with anti‐SAA antibody C‐1007. Note a single reacting species of ∼ 12 kDa in several but not all lanes. No smaller species were detected. Seventy‐five mcg (±15%) protein added to each lane.

Figure 1 shows a western blot of human colostrum samples reacted with antibody C‐1007. A band migrating at approximately 12 kDa is seen. Higher or lower molecular weight species were not detected. Levels of reactivity differed among the 10 specimens but similar migrating bands were present in 7 of the 10 specimens studied. Low‐molecular weight species were not detected.

**Figure 2:**
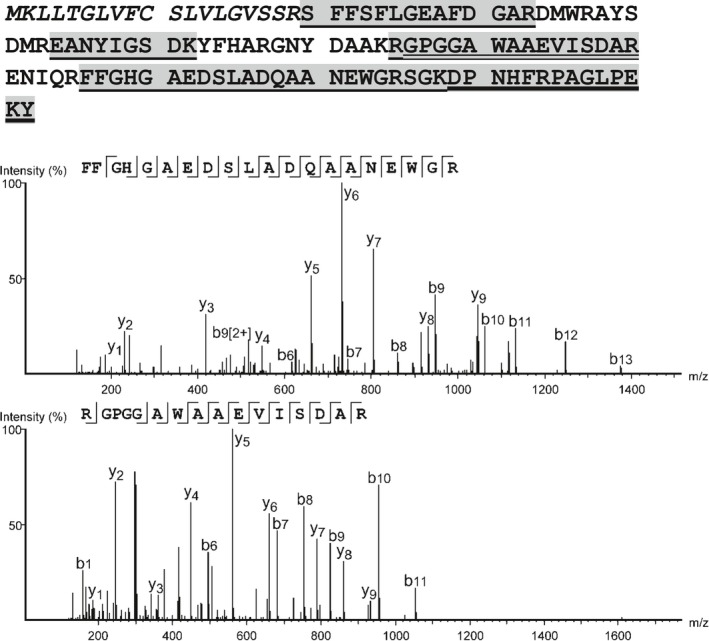
Amino acid sequence analysis. LC–MS/MS analysis identified 73% of the 104 aa of the secreted SAA1 sequence (top – aa of the signal sequence are shown in italics). The spectrum identifying FGHGAEDSLADQAANEWGR (middle) distinguishes the protein from other SAA classes. The RGPGGAWAAEVISDAR peptide (bottom) suggests that SAA1.5 natural variant (V70→A and A75→V) is the present allele. The search results are presented in PEAKS 7.0 with matched fragment ions (mass error < 0.01 Da) highlighted to distinguish b‐ions and y‐ions, the precursor mass error was below 5 ppm for all peptides.

Protein sequences were obtained using mass spectrometry as described. Polypeptides corresponding to human SAA1 based on recognized polymorphisms were identified as shown in Fig. 2. No sequences corresponding to SAA3 and/or containing the signature TFLK N‐terminal aa sequence, were found.

All SAA‐reacting species migrated as a single band of approximately 12 kDa. Sequence data identified SAA1 in this band. The molecular weight predicted by migration is consistent with that for SAA1, based on its 104 aa sequence (an 18 aa signal sequence is removed prior to secretion). Significantly, no polypeptides corresponding to SAA3 were identified. Based on these data, we conclude (a) SAA1 is present in some human colostrum samples, (b) neither a 12‐kDa species corresponding to SAA3 nor a short, milk‐specific SAA isoform was detected in these samples. The amount of SAA‐reactive species differed among the specimens but, to preserve anonymity, the precise timing of collections is unknown (although it reportedly differed due to clinical factors). Colostrum specimens in this study were obtained only from mothers following full‐term deliveries and without a history of corticosteroid treatment. Studies by McDonald *et al*. 14 showed that SAA was absent in mature milk; its production was consistently limited to colostrum possibly explaining the absence of SAA in three of our samples (which may have been collected later in the postdelivery period).

## Discussion

Knee *et al*. 17 using the same antibody (C‐1007) in an ELISA noted varying levels of reactivity in colostrum specimens. However, they did not present gel migration data or specifically identify the reacting protein species. The C‐1007 antibody was developed as a monoclonal antibody specific for SAA 18, and cross‐reactivity between all members of the SAA protein family occurs due to extensive sequence conservation. Thus, an ELISA using this antibody cannot distinguish among SAA family members.

**Figure 3:**
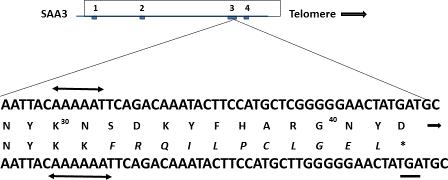
Nucleotide sequence of human SAA3 (below) below vs. cow (above) showing the single A nt insertion and resulting stop codon (TGA, underlined). The novel aa caused by the frameshift are shown in italics. The premature termination of the human protein at aa 42 is indicated although this protein has not yet been detected, see text.

Although it was suggested that the human SAA3 locus represents an unexpressed pseudogene 19, that report did not evaluate breast tissue or secretions. Larson *et al*. 16 found SAA3 mRNA expression in human mammary gland cell cultures (both MCF‐7 and T47‐D) following exposure to prolactin or LPS. The transcripts were appropriately spliced and contained an open reading frame corresponding to a 60 aa precursor protein containing the 18 aa signal polypeptide recognized in other SAA proteins and a mature 42 aa protein containing the TFLK sequence unique to SAA3. An (A) insertion at nt 204 of the cDNA led to a frameshift creating an early terminal codon but RACE extension showed that the transcript contained a 406 nt 3′ UTR rather than the 145 nt 3′ UTR predicted for a fully open reading frame 13. The position of the AAUAAA polyadenylation signal in the transcript corresponded to the earlier prediction 13. Thus, the human SAA3 gene can be transcribed (a translation product was not sought in that study). As shown in Fig. 3, the TGA stop codon in this mRNA is 51 nt 5′ to the 3′ splice site, making it likely that this transcript will succumb to nonsense‐mediated decay as predicted by Nagy and Maquat 20. Our efforts to identify a short, 42 aa, protein were unsuccessful – consistent with these observations. Comparing putative SAA3 sequences across multiple genomes shows this additional nucleotide in human, chimp and bonobo, preventing synthesis of full‐length SAA3 in these three species (see Fig. 3). The full‐length reading frame remains intact in murine, bovine, equine and ovine species, however, consistent with this distinction and the earlier report 14.

The human SAA1 gene contains an intact upstream glucocorticoid responsive element (GRE) and dexamethasone has been shown to increase its responsiveness to cytokines. Dexamethasone exposure alone did not lead to SAA1 transcription and the combined effect was appropriately blocked by GRE antagonist RU‐486 21. In contrast, in THP‐1 monocytes 22, aortic smooth muscle cells 23 and KB oral epithelial cells 24, dexamethasone alone can induce SAA transcription (without cytokines). However, prolactin responsiveness – which clearly stimulates SAA3 expression in bovine and human mammary cells 16, 25, 26 – has not been studied in other human tissues. Thus, despite apparent preservation of prolactin responsiveness in human breast epithelium *in vitro*, synthesis of full‐length mature SAA3 protein likely does not occur in humans due to a premature stop codon and nonsense‐mediated mRNA decay.

Studying bovine, ovine and caprine colostrum, McDonald *et al*. 14 consistently found an SAA species whose sequence(s) corresponded to that predicted for SAA3 in humans 13. All sequences shared distinct N‐terminal regions with a TFLK tetrapeptide that is not present in SAA1 or SAA2. Their work showed that TFLK alone could increase expression of MUC3 mucin in HT29 cells 15, 27, suggesting that this well‐conserved protein in colostrum could have a protective role in minimizing intestinal colonization and/or contamination in newborns. More recently, Tashiro *et al*. 28 showed MUC2 mucin mRNA in murine colonic epithelium CMT‐39 cells in response to exposure to aa 1‐36 of SAA3 or intact SAA3 but not SAA1.

Molenaar *et al*. 29 found SAA3 mRNA localized to restricted populations of bovine mammary epithelial cells and expressed at a moderate level in late pregnancy, at a low level through lactation and at high levels during involution and inflammation. They reported antibacterial activity of the mature protein against *Escherichia coli*,* Streptococcus uberis* and *Pseudomonas aeruginosa*, and hypothesized a role in protecting the neonatal gastrointestinal tract.

By contrast, a definitive role for either SAA1 or SAA2 (the serum APR constituents) has not been unequivocally established despite striking evolutionary conservation of both genes and proteins and their prominence in APR biology. Our finding, SAA1 in human colostrum implies that human newborns will be exposed to it during nursing and that this APR protein could be involved with intestinal physiology during the early neonatal period.

Recent studies have increased interest in the role(s) of SAA in gastrointestinal physiology. Ivanov *et al*. 30 reported that IL‐17‐secreting, Th17 cells accumulated in the lamina propria of the murine intestine as a specific consequence of noninvasive segmented filamentous bacteria (sfb) adhesion. Sfb adhesion to intestinal epithelial cells (not simply colonization) led to transcription of SAA1 as the most prominent upregulated species. Adding SAA1 to cocultures of dendritic and naïve CD4^+^ T cells induced Th17 cells in a concentration‐dependent manner. Atarashi *et al*. 31 proposed that actin reorganization in epithelial cells secondary to sfb adhesion led to elevated C/EBPδ expression which they proposed interacted with 2 DNase hypersensitive sites 3′ to the SAA1 gene. C/EBPδ levels were upregulated in sfb‐colonized mice where they emphasized the need for direct contact between the cells and sfb. Sano *et al*. 32 noted that IL17A production was greatest in the ileum and identified type 3 innate lymphoid cells (ILC3) which, following sfb adhesion, secreted IL22. This response then led to SAA1 production by epithelial cells through a Stat3‐dependent mechanism. The SAA1 effect on Th17 cells appeared to resemble cytokine activation.

These observations add to accumulating evidence for important role(s) of SAA proteins in both development and possibly maturation of intestinal defense and immune responses. The evolutionarily conserved presence of SAA proteins in colostrum establishes a potential role for these cytokine‐like proteins in neonatal host defense during a critical, early postnatal period and suggests possible therapeutic application(s) in conditions where exposure to these molecules may be limited (e.g. prematurity or malnutrition). In humans, finding SAA1 in colostrum in place of SAA3 as found in other mammals can be understood at the nt level in evolution (see Fig. 3). However, this implies a significant, ancient change in control and expression of SAA1 within this well‐conserved gene and protein family as well as a possible, novel, biologic role. Further study should clarify the likely important downstream physiologic effect(s) in more detail, particularly as they relate to neonatal health and development.

## Author contributions

NR obtained informed consent and collected all specimens. GS wrote the manuscript and performed protein studies in coordination with NZ. SK and RC performed mass spectrometry. CCT analyzed DNA databases. TM supplied antibodies and interpretive guidance.
